# Using optical coherence tomography to assess luster of pearls: technique suitability and insights

**DOI:** 10.1038/s41598-024-62125-9

**Published:** 2024-05-15

**Authors:** Yang Zhou, Lifeng Zhou, Jun Yan, Xuejun Yan, Zhengwei Chen

**Affiliations:** 1 School of Information and Electronic Engineering, Zhejiang University of Science and Technology, Hangzhou, 310023 Zhejiang China; 2 School of Innovation and Entrepreneurship, Zhejiang University of Science and Technology, Hangzhou, 310023 Zhejiang China; 3 Zhejiang Fangyuan Test Group Co., Ltd, Hangzhou, 310013 Zhejiang China

**Keywords:** Bioinformatics, Imaging, Sensors and probes, Optical spectroscopy, Applied optics

## Abstract

Luster is one of the vital indexes in pearl grading. To find a fast, nondestructive, and low-cost grading method, optical coherence tomography (OCT) is introduced to predict the luster grade through the texture features. After background removal, flattening, and segmentation, the speckle pattern of the region of interest is described by seven kinds of feature textures, including center-symmetric auto-correlation (CSAC), fractal dimension (FD), Gabor, gray level co-occurrence matrix (GLCM), histogram of oriented gradients (HOG), laws texture energy (LAWS), and local binary patterns (LBP). To find the relations between speckle-derived texture features and luster grades, four Four groups of pearl samples were used in the experiment to detect texture differences based on support vector machines (SVMs) and random forest classifier (RFC)) for investigating the relations between speckle-derived texture features and luster grades. The precision, recall, F1-score, and accuracy are more significant than 0.9 in several simulations, even after dimension reduction. This demonstrates that the texture feature from OCT images can be applied to class the pearl luster based on speckle changes.

## Introduction

Pearls and their products are popular gems because of their natural characteristics. The yield of Chinese pearls is dominant worldwide, and more than 90% of the products are from the Chinese jewelry industry^1^. The price of a pearl depends on its color, shape, luster, surface perfection, and more, and the price is highly related to grading based on the above index. Therefore, pearl grading is paid close attention. However, it still relies on manual operation. Manual inspection is time-consuming, and the grading errors are due to subjective deviation. Hence, the fast and nondestructive grading method has been taken as the critical point.

Machine vision has the advantages of accuracy, consistency, repeatability, and low cost. Cao et al. proposed a method for automated shape grading of pearls by the ratio of the long axis to the short axis of the pearl^2^. Zhou and Ma found defects in pearls based on the shape and texture features of defect regions^3^. Vigorelli et al. instigated the distinction between natural and cultivated pearls by X-ray micro-tomographic and electron microscope analysis^4^. Gordon et al. measured the nacre thickness of round pearls by micro-radiography^5^. Zhu et al. presented a new real-time method to measure and grade the pearl color using a CCD camera^6^. Nevertheless, the electron microscope is not a nondestructive method, and after the X-ray radiation, the color of the pearl fades, leading to value loss^7^.

In recent years, spectroscopy-based methods have been introduced to pearl grading, the reflectance of pearls and their peak was recorded, and then the pearl color was classified^8^. The decaying rate of the fluorescence spectra was related to the nacre characteristic and disclosed the type of treatments^9^. The UV-visible spectra were used as input to the artificial neural network, and the origin, species of the mollusk, color donor, possible color enhancement, and donor color were predicted by a trained network^10,11^. With the help of a hyper-spectral camera, the spatial information combined with spectra was acquired, and feature extraction and quantification approaches were used to predict the color, glossiness, and thickness of nacre^12^.

Luster, sometimes called glossiness, is decided by the combined effect of specular and diffuse reflection at the visible band^13^. The luster of pearls could depend on the different CaCO_3_ phases in their structures, and luster is one of the most important indexes for pearl grading. Even if it was measured or evaluated by UV-Vis spectroscopy by getting the color scales from spectra, the roundness of the surface causes an unstable measurement compared to the flat surface^14,15^. Due to the solid specular reflection on the surface of pearls, resulting in CCD saturation, the traditional machine vision approach cannot accurately evaluate glossiness. Hence, the industry’s nondestructive and rapid perdition of pearl luster is still a technical challenge.

Optical Coherence Tomography (OCT) can get a depth-resolved image of the pearl surface and already has many applications for peal inspection. The thickness measurement of nacre is a typical successful case, and OCT has become the national standard in China for the index of nacre. With the help of a high penetration depth device, the nucleus can also be observed through light penetrating the nacre. At the same time, it is easy to classify pearls into beaded and non-beaded categories. Meanwhile, cracks, crevices, or blemishes in the nacre and nucleus were detected^16–19^. Based on the above feature of OCT, our research group has applied it to inspect the internal defect of pearls by proposing a fully automated algorithm for defect classification^20^. The spectroscopic OCT allows spectroscopic measurements at the subsurface range of pearls, and it was used to classify authentic and artificial pearls in the NIR spectral range^21^. To overcome the drawbacks of 2D images or mechanical methods, our team also proposed an automated grading method of pearl roundness based on 3D OCT data and designed a new digital rating index for roundness classification^22^. The polarization-sensitive optical coherence tomography (PS-OCT) was used for pearl classification, and the difference between real and fake pearls was found in the Mueller matrix figured out from the OCT image^23^. By performing connected region labeling, fresh and saltwater pearls were classified. Moreover, the internal defects in pearls were detected by reviewing the M00 element chart^23^. OCT has become an effective method for monitoring micro-structure change and has more application prospects in the pearl industry.

Many automated image processing methods have been proposed for different OCT applications. We aim to relate the feature extracted from the OCT image to the luster variation. However, there is no inevitable correlation between changes in the cluster and morphology. The luster feature belongs to one kind of optical property. Different CaCO_3_ phases of pearl cause contrast or refractive index (RI) differences, which correlate with the underlying luster characteristic. For morphological features, speckle is often regarded as noise. Nevertheless, for optical features, the speckle may contain information on nacre structure. Schmitt et al. proved that the ‘inherent’ speckle is consistent, located in the same region in repeated OCT images, and regarded as a specific aggregate texture^24^, it manifests optical features as an optical signature based on structural changes. To quantitatively analyze these signatures, objective features were extracted as a digital index to make the decisions for luster level, among which texture features provide a quantitative evaluation to speckle patterns with many successful cases in the medical area^25^. Textures in pearl OCT images are not uniform because of grayscale variation. The previous research findings on texture have inspired us to believe that a combination of texture descriptors might be a more useful tool to reveal the change of speckles to the luster.

This study aims to (1) investigate the capability of OCT to classify the luster index of pearls accurately and (2) propose a flowchart for extracting different texture features from OCT images and aching automated classification of pearls based on luster. The study’s results will supplement automated pearl grading techniques for academic and industry applications.

## Results

**Figure 1:**
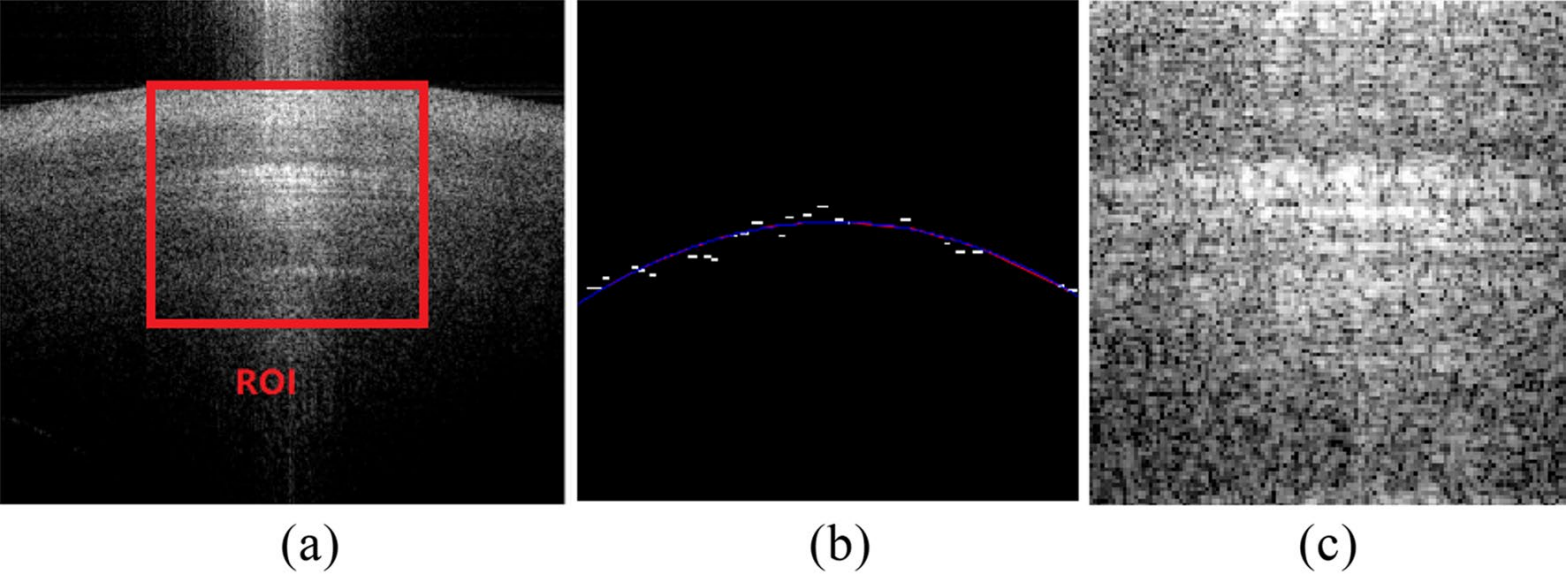
(**a**) the original OCT image of pearl (**b**) rough edge and fitted edge (**c**) ROI after segmentation.

The typical OCT images of pearl were shown in Fig. 1, and the ROI region after flattening and segmentation was also displayed in Fig. 1c. From Fig. 1a, we found it difficult to discriminate the luster of pearls by native eyes, and the texture features were worth investigating for the speckle patterns. From Fig.1b, the fitted edge coincided with the actual surface of the nacre, and then the ROI, after flattening and segmentation, was the demanded target for the following texture analysis.

The data set of pre-processed pearl OCT images was split into train and test groups by the ratio of 8/2, resulting in the two groups being 92 and 24, respectively. All 237 features were used for building the SVMs and RFC models, while the hyperparameters of two classifiers were randomized and searched under 50,000 iterations. As a result, the optimal kernel, gamma, and C values of SVMs were ‘rbf,’ 0.002, and 2.6, while the optimal criterion, max features, and number of RFC were ‘gini,’ 10 and 25, respectively.

**Figure 2:**
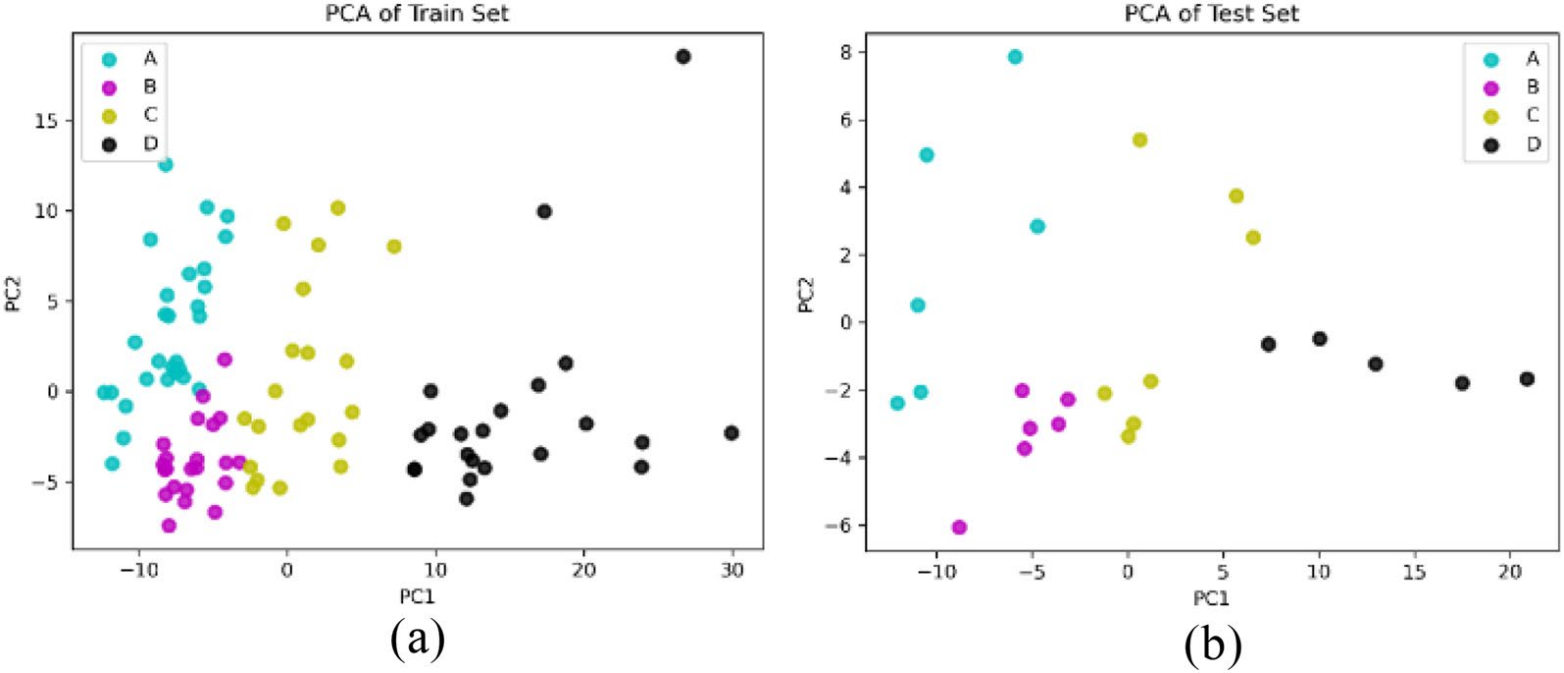
The distribution on the first two principal components (**a**) train set (**b**) test set.

For another prospect to feature selection, principal component analysis (PCA) was applied to get the main principal components or directions in the feature space that account for the most variance, and the high dimensional data was projected to low dimensional space, which was considered as dimension reduction. The scatter of all the samples on the first two principal components that explained the most variance was plotted in Fig. 2, in the components space, different luster groups were clustered at different positions, and it had the potential to find an interface among the groups. In the prediction, the scores of the first 6 components were used as the input of SVMs and RFC, where those 6 components accounted for more than 90% variance. Table 1 shows the luster grading result from two classifiers based on all 237 features and scores after PCA. The experiment under each setting was run ten times, and the average, maximum, and minimum values of Precision, Recall, F1-Score, and Accuracy were listed.

**Table 1:** The prediction results of SVMs and RFC.

Experiment	Precision			Recall			F1-score			Accuracy		
	Max	Min	Avg	Max	Min	Avg	Max	Min	Avg	Max	Min	Avg
SVMs_train_all	1	0.98	0.99	1	0.98	0.99	1	0.98	0.99	1	0.98	0.99
SVMs_test_all	1	0.92	0.95	1	0.90	0.94	1	0.89	0.93	1	0.92	0.94
RFC_train_all	1	1	1	1	1	1	1	1	1	1	1	1
RFC_test_all	1	0.90	0.94	1	0.91	0.95	1	0.90	0.94	1	0.92	0.94
SVMs_train_PCA	0.99	0.95	0.97	0.99	0.95	0.97	0.99	0.94	0.97	0.99	0.95	0.97
SVMs_test_PCA	1	0.92	0.97	1	0.90	0.96	1	0.91	0.96	1	0.92	0.96
RFC_train_PCA	1	1	1	1	1	1	1	1	1	1	1	1
RFC_test_PCA	1	0.91	0.97	1	0.91	0.96	1	0.91	0.96	1	0.92	0.97

From Table 1, the accuracy of each prediction reached more than 90%, indicating that the texture features were related with the luster changes. The performance of RFC was slightly better than that of RFC because the SVMs aimed to find a classification hyperplane causing higher sensitivity to feature data distribution. The RFC established a multi-level decision tree to achieve optimal generalization performance that did not require outlier handling and had better adaptability under different distributions. After dimension reduction by PCA, the predictive performance of SVMs was slightly decreased. Nevertheless, the predictive performance of RFC remains essentially unchanged, indicating that the RFC had better generalization ability. Overall, there was no significant difference in predictive performance between the two classifiers, and the experiment results proved the feasibility of applying OCT technology to pearl luster grading.

**Figure 3:**
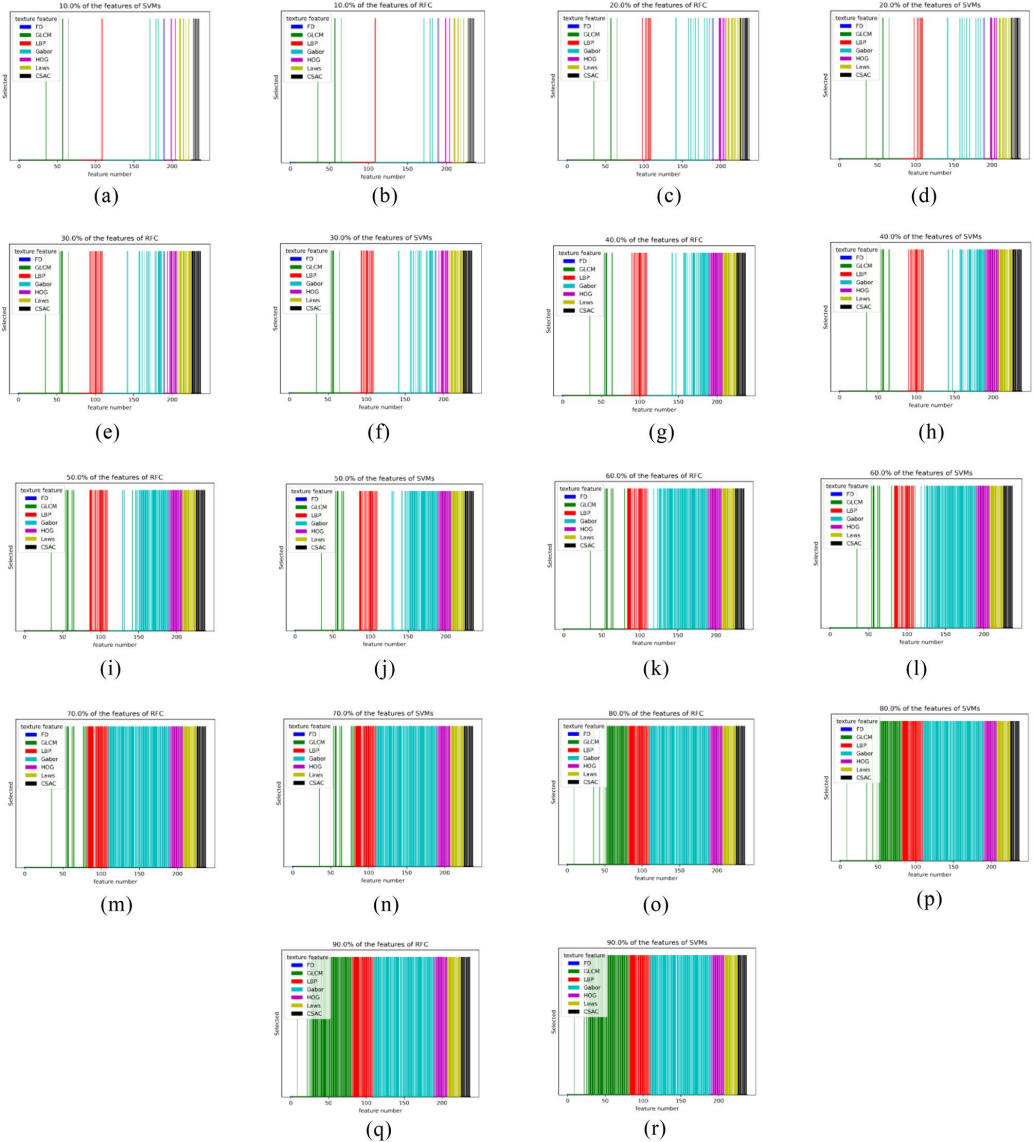
The result of feature selection. 10–90% of features were selected for SVMs and RFC.

Following this, the sequential feature selector removes features individually to find the feature contributions. The mask of selected features was plotted and shown in Fig. 3. From Fig. 3a–r, 10–90% of all the features were select classifier scores, and the distribution of feature type was illustrated.

The FD value almost had no contribution to luster classification during the feature selection. However, the rest of GLCM, LBP, Gabor, HOG, Laws, and CSAC were related to the luster changes of the pearl OCT image. The amount of LBP, GLCM, and Gabor features was reduced rapidly, and the amount of HOG, Laws, and CSAC features showed a slight decrease to illustrate that the HOG, Laws, and CSAC played an essential role in the classification of speckle patterns. The roles of LBP, GLCM, and Gabor existed when 10% or 20% of features were selected, and some of them still affected classification.

## Discussion

At the point of a fast and nondestructive method for pearl luster grading, we only found that UV reflectance spectroscopy combined with a neural network was used to predict the luster of pearl samples^10,11^. In the report, only a few samples were tested, and it only showed that no predictive model was 100% accurate for luster grading. It was the first time we had used the OCT technique for pearl luster grading, and our research provided a new idea for classifying pearl glossiness.

Due to the characteristics of OCT devices and the material themselves, speckle has always existed, and it is often considered a type of noise that is routinely removed^26^. However, our research is from another perspective. The speckle contains information of material relevance and can be divided into two categories: stochastic speckle and inherent speckle. The stochastic speckle is random and caused by multiple scatter of light. It was removed by averaging OCT scans during the image acquisition. By contrast, inherent speckle was caused by multiple scattering effects when light penetrated through the nacre of pearl, which was related to the material characteristic and was consistent in repeated tests. Cumulatively, the inherent speckle generated the specific texture features that might be correlated with luster changes. The texture can reflect subtle changes in optical properties and provide a potential tool for pearl luster grading, which was the fundamental idea of our research.

The speckle pattern was digitized with the help of texture features, and the machine learning algorithms achieved satisfying accuracy. The core investigation opened a new technique view for pearl grading and made up for the deficiency from other additional methods described in the introduction section. Compared with our previous study, the OCT technique performed an alternative way to grade the pearl luster. For the RBG camera, with the limitation of the dynamic range, it was not easy to calibrate the amount of light reflected from the pearl surface. Similarly, any spectroscopy-based measure also had saturation, and the luminous flux was affected by some factors, such as shape, variety, treatment, etc. Nevertheless, the OCT image provided a direct approach to reveal the subsurface structure, essentially, the tomographic image was constructed by light changes, which correlated more with glossiness/luster. Recently, OCT has been introduced to the pearl industry for nacre thickness measurement, treatment analysis, and inter-defect inspection. Unfortunately, finding a relation between the OCT image and pearl luster was difficult, which prompted us to use texture features in luster grading and expanded the application scope of OCT technology.

The OCT device was expensive a few years ago, making it only affordable in the research scenarios. Nevertheless, the low-cost OCT device gradually emerged and was promoted into the pearl industry. Compared with the traditional 2D machine vision, we used 3D tomographic images to characterize the luster of pearls by which more features were extracted. It took 2 S for image workflow in a routine laptop, which was acceptable in an actual application. Also, to increase the speed of parallel processing based on GPU devices, computational efficiency would be improved based on increased equipment costs.

In recent decades, deep learning networks have been widely applied in classification tasks, and the network needed large amounts of known samples for training^27^. We also used our feature data set to train a deep network and found that the perdition accuracy of the trained net for the test set was over 95%. Compared with SVMs or RFC, the performance was almost at the same level, and the net might have been severely over-fit. Our feasibility study did not have enough samples due to the limited funding, so we were unsure if the deep network was suitable for our study.

We also tried to use some new methods for small sample clarification. With very little supervision from labeled data, few-shot learning was one of the typical ones^28^. In the original, the few-shot classifier could recognize new categories from very few labeled examples, while a few examples trained classifiers in each class, and the tanning procedure was decomposed into the meta-learning phases where transferable knowledge was learned based on different strategies. One of the few-shot classifiers was introduced to our luster grading based on those texture features. In detail, we modified the classic two-branch Relation Network (RN) by canceling the embedding module and directly inputting the extracted texture feature to perform a few-shot clarification^29^. We constructed the 4-way 5-shot learning (4 grades and 5 samples in each grade) with 5 query images per call for each iteration of the training, and an independent test set was randomly selected for validation. Unfortunately, the prediction accuracy only reached a little more than 90%, worse than that of SVMs or RFC.

However, deep learning methods required many labeled training samples, and acquiring labeled samples took much work. The few-shot clarification was designed because the testing domain categories differed from the training domain sample categories. In contrast, the training domain images often had only a few labeled samples. In contrast, our application’s training and testing domain had four of the same grades/categories. Hence, the critical approach used in our trying was meta-learning. Classifying accurately with only a few labeled samples by multiple mete learning steps was extremely difficult. From another perspective, although the classification accuracy was slightly lower, the effectiveness of the proposed method for pearl luster classification has been verified through the comparison of multiple methods.

## Methods

### Pearl sample and image acquisition

A low-cost spectral domain OCT (Lumedica OQ Labscope) with a central wavelength of 840 nm was used. The axial resolution and lateral resolution were 7 μm and 15 μm, respectively. The refractive index of the pearl was around 1.53 on average, and the lateral resolution was changed to about 11 μm in the pearl sample. Then, the B-scan images were acquired with a resolution of 512 × 512 pixels. Total of 116 OCT images were acquired from the pearl samples provided by Zhejiang Fangyuan Testing Group Co. Ltd. The pearl samples comprised seawater-nucleated pearls, freshwater-nucleated-free pearls, and freshwater-nucleated pearls. The seawater pearls included Nanyang white pearls, Nanyang gold pearls, and Japanese Akoya pearls, and the freshwater pearls included Zhuji freshwater nucleated pearls and Edison nucleated-free pearls. All of them were typical products in the Chinese or World market. The pearls’ luster was divided into four groups (A, B, C, and D group), and each of the four labels was assigned to one OCT image. The criteria of classification were the National recommended standards of China (Cultured pearl grading, GB/T 18781-2023) based on the brightness, sharpness, and uniformity of reflection. The varieties of these samples were distributed among seawater and freshwater pearls on behalf of typical Chinese pearls.

### Automated target location

The segmentation step was to locate the central part of the nacre layer of pearl as the target for cluster analysis. However, the size and roundness of the pearls were diverse, causing uncertainty about the location of the nacre, so background removal, flattening, and segmentation were suggested. The μ+2 × σ value, where μ and σ were the mean and variance of the grayscale of the top 30 rows, was used as a hard threshold for denoising. Then, the Canny edge operator was performed to get the rough edge between the nacre layer and the background. Following, the rough edge was fitted by the polynomial, while the pixels above the fitted edge were removed as the background. Then, the pixels in each column of the pearl target were shifted up and down to make all the points of the fitted edge lay on a horizontal line. Finally, the region with 128 × 128 pixels at the middle upper part of the flattened image was cropped as the region of interest (ROI) for texture analysis.

### Digitalize luster index

The OCT image’s texture was formed by pixels with different greyscale levels, there were different descriptors for characterizing those spatial relationships (listed in Table 2). Each target was characterized by a heterogeneous feature array of 237 elements that provided a potential relationship with luster change.

**Table 2:** The texture features used in the following investigation.

Feature type	Indexes
Fractal dimension (FD)	FD value [1]
Gray level co-occurrence matrix (GLCM)	Means of the map [2–41] Standard deviations of the map [42–81]
Local Binary Patterns (LBP)	Means of LBP cells [82–95] Standard deviations of LBP cells [96–109]
Gabor filters	Means of each combination [110–149] Standard deviations of each combination [150–189]
histogram of oriented gradients (HOG)	Bin average of all cells [190–197] Bin standard deviations of all cells [198–206]
laws texture energy (LAWS)	Means of filtered image [207–215] Standard deviations of filtered image [216–224]
center-symmetric auto-correlation (CSAC)	Means of 6 CSAC values [225–230] Standard deviations of CSAC values [231–237]

#### CSAC

The speckle pattern in the OCT image varied locally, and the center-symmetric auto-correlation (CSAC) method could reflect the local intensity variations caused by local structure variation^30,31^. The definition of CSAC was based on the relationships between each pixel and its 3 × 3 neighboring pixels. Six CSAC indicators were figured out for each OCT image, including grayscale texture covariance (SCOV), local variance (VAR), between-pair variance (BVAR), within-pair variance (WVAR), variance ratio (SVR), and normalized SCOV (SAC), which had different focuses on gray-level variation or intensity variance with the advance of invariant under linear gray-level shifting. For each 3 × 3 neighboring domain, there were 6 CSAC values, and the means and standard deviations of SCOV, VAR, BVAR, WVAR, SVR, and SAC of all the domains in one ROI were contributed to the feature array with 12 elements.

#### FD

The fractal dimension (FD) was one of the critical indicators for the fractal. It strongly correlated with roughness, and there were several methods for figuring out the FD. The traditional one, the differential box-counting method (BCM) by the number of boxes covering an ROI, was used in our experiment^32^.

#### Gabor

Gabor filters were widely applied in texture-related applications and could serve to feature the pattern of speckles as the indicator. It had the advantages of invariance to rotation, translation, or scale. It was fit for noise-like deformation, which had potential relationships with pearl luster change. Therefore, a combination set of 5 scales of wavelengths and eight orientations of 2D Gabor filters was applied to ROI^33^. The wavelength of the carrier was in the range of [2 4 8 10 12], while the filter’s orientation in degrees was in the range of [0 45 90 135 180 225 270 315]. Each combination’s means and standard deviations were derived, and 80 Gabor features were obtained.

#### GLCM

The gray level co-occurrence matrix (GLCM) discovered the relationship between adjacent grayscale levels^34^, and probability density functions were calculated based on its spatial histogram of the OCT image, the entropy, energy, correlation, and inertia (contrast) at the distance of 1-10 between grayscales and 0◦, 45◦, 90◦, and 135◦ direction were extracted for describing the contrast, grayscale distribution. A total of 40 combinations of GLCM feature maps were figured out, and each map’s means and standard deviations were used to create a feature array with 80 elements.

#### HOG

The histogram of oriented gradients (HOG) was generated by gradients at different regional orientations. The gradients in all cells of all blocks were calculated, and then the HOG feature was figured out from orientation-based histogram bins, which were invariant to scale rotation or translation operations^35^. The ROI with 128 × 128 pixels was regarded as one block, and the size of the cell and the number of orientation histogram bins were set at [4,4] and 9, respectively. A total of 1024 histograms was obtained, and then the bin average and bin standard deviations of all the cells were the input of the feature array with 18 elements.

#### LAWS

A set of different convolution kernels which could reveal different texture properties defined as laws texture energy (LAWS), where these 2D kernels were derived by multiplying the 1D masks with fixed window size (L5L5 was excluded), such as L3=[1 2 1], E3=[− 1 0 1], S3=[− 1 2 − 1], L5=[1 4 6 4 1], E5=[− 1 − 2 0 2 1], S5=[− 1 0 2 0 − 1], and R5=[1 − 4 6 − 4 1], where L, E, S, R masks were related to grayscale, edge features, spots and ripple patterns, respectively^36^. The 9 mask combinations of L5E5/E5L5, L5S5/S5L5, L5R5/R5L5, E5S5/S5E5, E5R5/R5E5, E5E5, S5R5/R5S5, S5S5 and R5R5 were selected, and the means and standard deviations of filtered image with 18 elements were generated.

#### LBP

Local Binary Patterns (LBP) encoded the local grayscale relationship of the neighboring pixels into a binary array^37^. The sensitivity of LBP to illumination was low, and it was one of the typical indicators for local patterns due to different configuration parameters and capture machines. The ROI was also regarded as an LBP cell and a wide range of parameters was studied, using several neighbors of 4, 8, 12, and 16 and a filter radius ranging from 1 to 5. There were 14 neighbor/radius combinations, including 4/1, 4/2, 8/1, 8/2, 8/3, 12/1, 12/2, 12/3, 12/4, 16/1, 16/2, 16/3, 16/4 and 16/5. The means and standard deviations of LBP cells with 28 elements were generated.

### Pearl grading based on its luster

A total of 237 features were considered as the input of support vector machines (SVMs), and random forest classifier (RFC), two of the typical supervised classifiers, were used to predict the luster level of the pearl. In order to avoid over-fitting, the type of kernel, corresponding kernel coefficient of ‘rbf,’ ‘poly,’ ‘sigmoid,’ regularization term for SVMs, the number of trees, and max features at each split for RFC were randomized searched by stratified 5-fold cross-validated to get those optimized parameters. The number of iterations was set at 50,000 in our experiment, enough to traverse a sufficient number of parameters within the setting range.

Sequential feature selection (SFS) methods evaluated seven kinds of features, and the SFS was running in backward mode. In each epoch, the sequential feature selector got the best features individually to form a feature set in a greedy style. The removal was based on the result from the cross-validation of the classifier. It started with all the features set and greedily removed the best features from a subset. The selection stopped when the desired number of features was acquired. The classifier’s performance was evaluated based on the confusion matrix, and then the F1 score, recall, precision, and accuracy were derived.

From another prospect, decomposition was another style to compress the data. The Principal Component Analysis (PCA) was used to decompose the feature matrix into a set of orthogonal components under maximum variance. The PCA was regarded as a transformer that learns components in its fit method from train feature data. It projected feature data on these components and reduced the dimension of the feature data. Our experiment’s feature set comprised 237 features projected on the six dimensions that explained the most variance. Hence, the dimension of classier input was reduced from 237 to 6, improving the speed of the prediction. Finally, the low dimensional data of the train set was used to train the SVMs and RFC, then the luster of the test set was predicted by the trained classifiers, and their performance was also compared.

## Data Availability

The datasets used and analyzed during the current study are available from the corresponding author (Y.Z.) upon reasonable request.
